# Prevalence of accelerometer-measured physical activity in adolescents in Fit Futures – part of the Tromsø Study

**DOI:** 10.1186/s12889-020-09171-w

**Published:** 2020-07-17

**Authors:** Sigurd K. Beldo, Bente Morseth, Tore Christoffersen, Peder A. Halvorsen, Bjørge H. Hansen, Anne-Sofie Furberg, Ulf Ekelund, Alexander Horsch

**Affiliations:** 1 Department of Community Medicine, UiT The Arctic University of Norway, Tromsø, Norway; 2 School of Sport Sciences, UiT The Arctic University of Norway, 9509 Alta, Tromsø, Norway; 3 Finnmark Hospital Trust, Alta, Norway; 4 Department of Health and Care Sciences, UiT The Arctic University of Tromsø, Tromsø, Norway; 5 Department of Public Health, Sport and Nutrition, University of Agder, Kristiansand, Norway; 6 Department of Microbiology and Infection Control, University Hospital of North Norway, Tromsø, Norway; 7 Department of Sports Medicine, Norwegian school of sport sciences, Oslo, Norway; 8 Norwegian Institute for Public Health, Oslo, Norway; 9 Computer Science Department, UiT The Arctic University of Norway, Tromsø, Norway

**Keywords:** Population-based cohort, ActiGraph GT3, Physical activity recommendations, Self-perceived health, School program, Socioeconomic status

## Abstract

**Background:**

Previous studies show large variations in physical activity (PA) levels among adolescents. However, the number of studies is limited and even fewer studies have assessed PA in adolescents by accelerometer devices. This study aimed to describe accelerometer-measured PA levels in adolescents in a population-based cohort in Northern Norway.

**Methods:**

In 611 students aged 16–17 years attending the Fit Futures Study, PA was measured by Actigraph GT3X for seven consecutive days. PA was expressed as total PA volume (counts per minute, CPM), time spent in intensity zones, steps per day, and fulfilment of WHO recommendation (i.e. accumulation of 60 min or more of at least moderate intensity PA per day). Potential correlates of PA such as sex, socioeconomic status, study program, self-perceived health, and PA variations by weekday versus weekend were also examined.

**Results:**

16% of the girls and 25% of the boys fulfilled current WHO-recommendations. Total PA volume (CPM) was higher in boys than in girls (353 (SD 130) versus 326 (SD 114) CPM, *p* < 0.05). PA levels differed with study program and increased with better self-perceived health, but were not associated with socioeconomic status. Both boys and girls were more active on weekdays than weekends (altogether; 350 (SD 124) versus 299 (SD 178) CPM, *p* < 0.05).

**Conclusions:**

In this cohort of adolescents, less than 25% of 16–17-year-old boys and girls fulfilled the WHO recommendations. The levels of physical activity in 16–17-year-old adolescents are similar to previous data reported in adults.

## Background

Insufficient physical activity (PA) is one of the leading risk factors for mortality globally [1, 2], and is associated with higher risk of non-communicable diseases (NCDs) [3–6]. Levels and patterns of PA seem to differ across the lifespan [7], and current literature indicates that PA levels are highest at the age of 6–9 years [7–9]. Studies indicate a 30% reduction in PA throughout adolescence from age 15 years [10] to age 20 years [11]. Moreover, a review of worldwide secular trends concludes that PA levels among adolescents are declining [12]. A recently published paper shows that this is a global phenomenon, independent of income levels of a country and cultural diversity [13]. PA as behaviour tends to track from adolescence to adulthood [14–17], and knowledge about PA levels and patterns in adolescents could help direct efforts and resources to prevent physical inactivity as adults.

There are different ways to measure PA, with different strengths and weaknesses. Questionnaires gives an insight in what kind of activity (behaviour) and can include types of activities not recognized by accelerometers. However, accelerometers are objective indicators of body movement (acceleration) and yield more precise measures of intensity, frequency and duration [18]. Most population-based studies of PA are based on self-reported data, which have been shown to overestimate PA [19] and therefore may yield crude and inaccurate estimates. To develop high quality evidence-based public health interventions, more precise PA estimates are warranted.

The use of devices such as accelerometers to measure PA is increasing, providing more accurate data on PA levels and patterns [20]. Device-based measured PA levels among adolescents indicate large variations, and existing studies report low compliance to PA recommendations [10, 20–23]. However, there is a paucity of data on accelerometer measured PA among older adolescents. The aim of this study was therefore to fill this gap, by describing accelerometer-measured PA in adolescents aged 16–17 years old in Northern Norway and to examine potential correlates of PA in this age group.

## Methods

### Study population and design

The Fit Futures Study (TFF) is a population-based cohort study of adolescents in Northern Norway and part of the population-based Tromsø Study [24, 25]. We used data from the Fit Futures 1 (TFF1), which was carried out from September 2010 to April 2011. All students in their first year upper secondary school, which is the 11th school year in Norway, were invited to participate. The data collection included questionnaires, clinical examinations, and blood samples. Altogether 1117 students from one urban and one rural municipality were invited, and 1038 (92.7%) participants attended, involving 8 different schools and 3 different study programs (general, vocational, and sports studies). The participants were recruited through the schools, and the examinations were conducted during a school day.

#### Participants without valid accelerometer data were excluded

The participants signed a written informed consent. Participants younger than 16 years of age signed with written permission from guardians and those aged 16 and above signed at the study site. The Regional Committee for Medical and Health Ethics has approved the study (2012/1663/REK nord).

### Data collection

The participants filled out an electronic health and lifestyle questionnaire including self-reported PA, self-perceived health (very bad, bad, neither good nor bad, good, excellent), and parents’ education as a proxy of socioeconomic status (SES) (don’t know, primary school 9 years, occupational high school, high school, college < 4 years, college 4 ≥ years) (Additional file 1). The parent with the highest education was regarded as “parents’ education”. Experienced technicians conducted a physical examination. Height and weight were measured following standardized procedures including light clothing and no shoes on an automatic electronic scale, Jenix DS 102 stadiometer (Dong Sahn Jenix, Seul, Korea). BMI was calculated as weight in kilograms divided by the squared height in meters and categorized into < 18 kg/m^2^ (underweight), 18–24.9 kg/m^2^ (normal weight), 25–29.9 kg/m^2^ (overweight) and ≥ 30 kg/m^2^ (obese). Study program (vocational, general studies and sports) was registered. At the end of the examination, the accelerometer was handed out. After 8 days the accelerometer was collected at school.

### Assessment and processing of physical activity data

Physical activity was assessed with the ActiGraph GT3X (ActiGraph, Pensacola, FL), recording accelerations in three axes (axial, coronal and sagittal). Trained technicians instructed the participants to wear the accelerometer on their right hip attached with an elastic band for seven consecutive days, and to remove the ActiGraph only for water-based activities and during sleep. The devices were initialized in ActiLife with sampling frequency 100 Hz and default filter was used to aggregate raw data into epochs of 10 s. Data were collected between 14:00 on the first day and until 23:58 on day eight. The first day of measurements was removed to reduce reactivity [26]. In accordance with other studies [27], measurements were included in the analysis if the participant had accumulated at least four days of ≥10 h per day of activity.

2Non-wear time was identified using a triaxial method described by Hecht et al. 2009 [28]. A minute was considered wear time if: either its value was > 5 vector magnitude units (VMU) CPM and there were at least 2 min > 5 VMU CPM during the time span of 20 min before and / or after this epoch, or its value did not exceed 5 VMU CPM, but both on the preceding, and on the following 20 min there were 2 or more minutes > 5 VMU CPM, otherwise as non-wear time. The ActiLife v6.13.2 software was used for downloading of accelerometer data (ActiGraph, LLC, Pensacola, USA), and further data processing was done with the Quality Control & Analysis Tool (QCAT). Prior to analyses in QCAT, the data was aggregated to epochs of 60 s. This was considered reasonable for the basic variables related to volume, intensity and duration of PA, and made our study comparable to other Norwegian studies [8–10, 29]. In this study, uniaxial data are presented for comparability with previous studies. Freedson uniaxial intensity cut-points were used to categorise time (min/d) into different intensity levels as follows [30]: Sedentary behaviour 0–99 CPM, light PA ≥100–1951 CPM, moderate PA ≥1952–5724 CPM, and vigorous PA ≥5725 CPM [31]. Moderate and vigorous PA were merged into moderate to vigorous PA (MVPA). Step counts are accumulated on a per-epoch basis and based on accelerometer data collected from the vertical axis [32].

PA was quantified as counts per minute (CPM) from the vertical axis. The following PA variables were extracted for use in this study: Accumulated minutes per day spent in the different intensity categories; mean number of counts per minute (CPM); percentage of the population fulfilling the WHO minimum recommendations of ≥60 min MVPA per day [33]; steps per day; and the percentage of participants accumulating ≥10.000 and ≥ 6000 steps per day. We chose 10.000 steps per day because this is a commonly used cut off value, and several studies have shown a correlation to fulfilment of activity recommendations of 60 min MVPA per day [34, 35]. On the other hand a cut off of 6000 steps per day has been associated with a sedentary lifestyle [35, 36].

### Statistical analyses

Differences in PA levels between girls and boys were analysed using Student’s t-test, and differences between weekday and weekend PA levels were analysed using paired-samples t-test. Differences in PA levels by SES, self-perceived health and study program were analysed using Fisher’s one-way ANOVA. In cases of unequal variances, Welch’s ANOVA was used. All analyses were performed using Statistical Package of Social Science (SPSS v. 25) and all values of *p* < 0.05 were considered statistically significant.

## Results

**Fig. 1:**
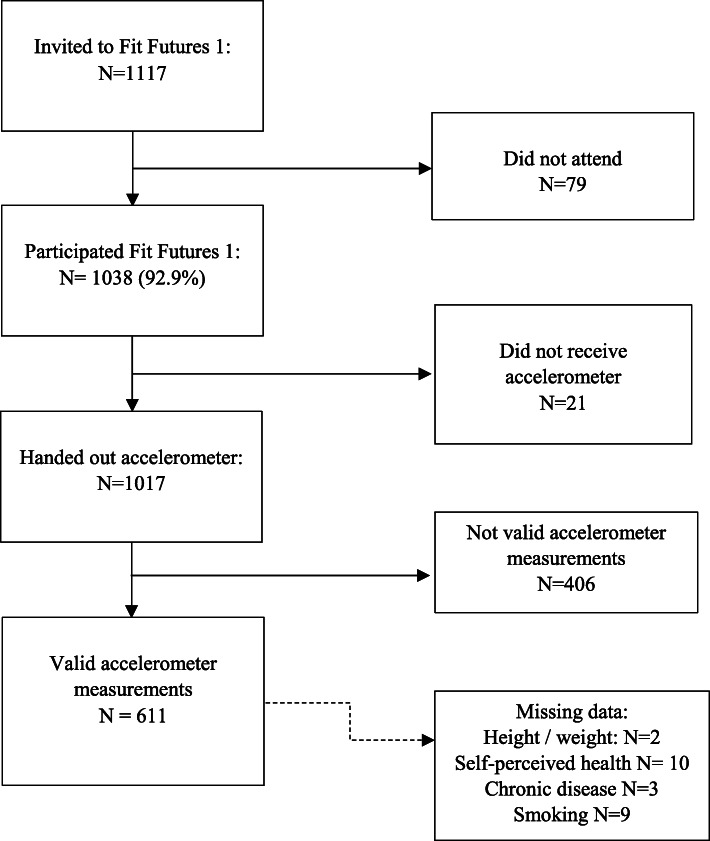
Participation in the Tromsø Study: Fit Futures 2010–11

In total, 611 participants had valid accelerometer measurements (Fig. 1).

The majority of the respondents were non-smokers and considered their health to be good or excellent (Table 1). About 30% reported one or more chronic diseases (in order of prevalence): Asthma (7.2%), allergic rhinitis (5.6%), migraine (2.5%), eczema (2.3%), Attention Deficit Hyperactivity Disorder (ADHD) (1.1%), others (all < 1.0%).

**Table 1:** Participant characteristics. The Tromsø Study: Fit Futures

	N (girls/boys)	All	Girls	Boys
Age, mean (SD)	611 (341/270)	16.3 (1.0)	16.4 (1.1)	16.2 (0.8)
Height, cm (SD)	609 (339/270)	170.3 (8.9)	165.0 (6.6)	176.9 (6.7)
Weight, kg (SD)	609 (339/270)	65.4 (13.8)	61.3 (11.7)	70.6 (14.4)
Body-mass index, kg/m2 (SD)	609 (339/270)	22.5 (4.1)	22.5 (4.0)	22.5 (4.2)
< 18.0	609 (339/270)	9.7%	7.7%	12.2%
18.0–24.9	609 (339/270)	69.4%	72.4%	65.6%
25–29.9	609 (339/270)	14.6%	13.2%	16.3%
≥30	609 (339/270)	6.1%	6.2%	5.9%
No chronic diseases	608 (339/269)	69.6%	66.9%	73.0%
Smoking	602 (335/267)			
Daily		3.8%	3.6%	4.1%
Sometimes		13.1%	14.0%	12.4%
Never		81.7%	82.4%	83.5%

In total, mean (SD) accelerometer wear time was 14.1 (1.14) hours per valid day (girls 13.98 SD 1.07 and boys 14.25 SD1.21, *p* = 0.053). Participants with valid accelerometer data did not differ significantly from those who did not wear an accelerometer with respect to sex, BMI, and self-perceived health (data not shown).

Participants spent 67% of the accelerometer wear time within the sedentary category, 28% in light intensity activities, 4.8% in moderate and 0.4% in vigorous activity (Table 2).

**Table 2:** Minutes in different intensity levels in 16/17-year-old boys and girls. The Tromsø Study: Fit Futures

	N	Sedentary activity (CPM 0–99)Mean (95% CI)	Light activity (CPM 100–1951)Mean (95% CI)	Moderate activity (CPM 1952–5724) Mean (95% CI)	Vigorous activity (CPM ≥5725) Mean (95% CI)
**All**	611	566.5 (560.8–572.1)	235.5 (231.2–239.9)	41.1 (39.6–42.7)	2.9 (2.6–3.3)
**Boys**	270	571.0 (561.5–580.5)	236.3 (229.0–243.7)	44.5 (42.1–46.9)	3.2 (2.6–3.7)
**Girls**	341	562.8 (555.9–569.7)	234.9 (229.6–240.1)	38.5 (36.6–40.4)	2.7 (2.3–3.2)

**Fig. 2:**
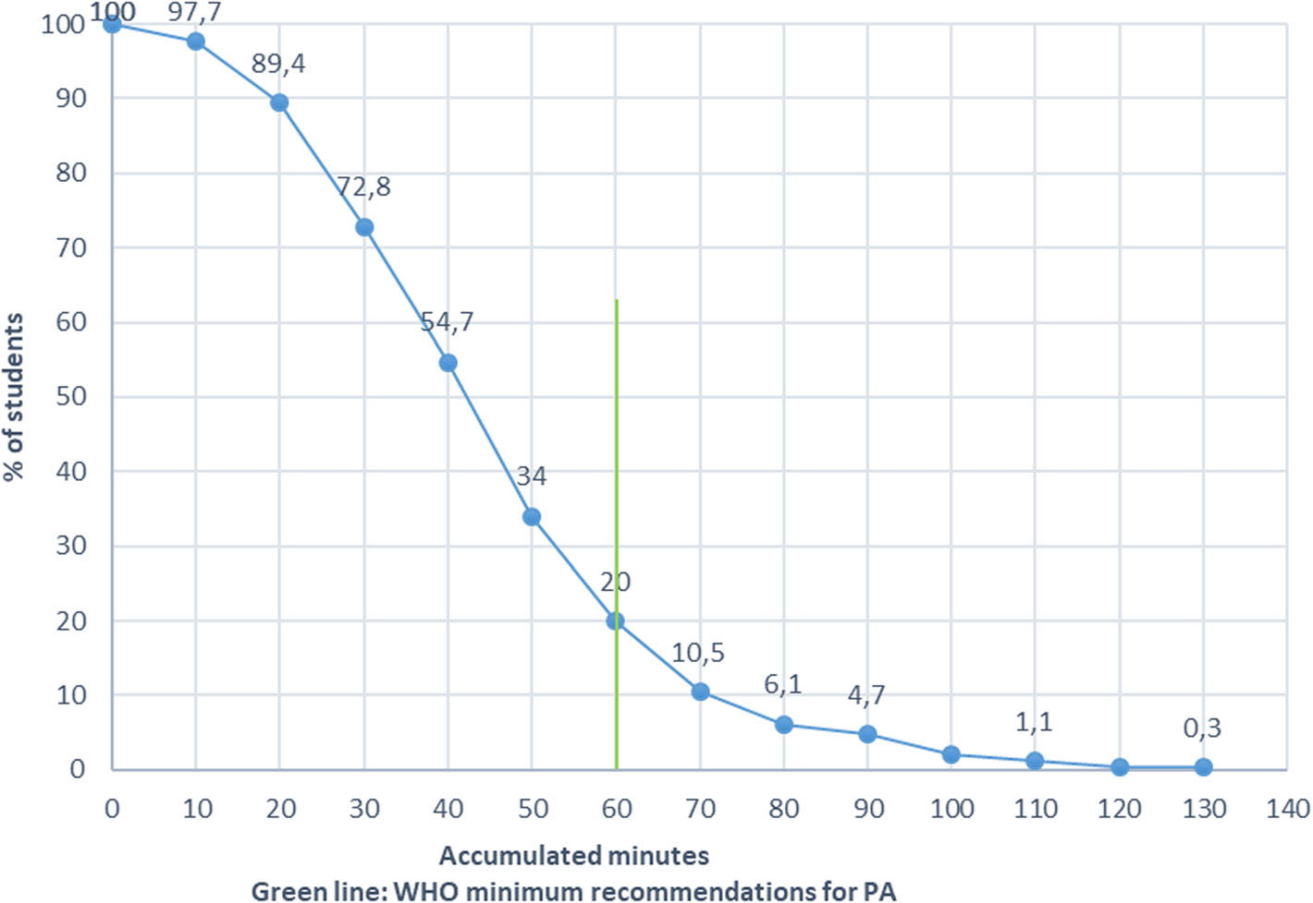
Distribution of mean minutes of MVPA per day in 16/17 years old boys and girls, The Tromsø Study -Fit Futures

Mean time spent in MVPA per day was 44.1 (SD 21.5) minutes. Boys spent 6.4 min more in MVPA than girls (95%CI boys 44.9–50.4, girls 39.1–43.4). There was no significant difference in MVPA between BMI groups. Both boys and girls who rated their self-perceived health as excellent accumulated more minutes of MVPA than all the other groups (*p* < 0.05, Table 3). Participants with parents’ education ≥4 years of college spent 8.8 min more in MVPA per day compared to participants with parents educated from vocational school (*p* < 0.05). Study program was associated with the amount of registered MVPA (p < 0.05), with sports-students presenting 79.5% more MVPA than vocational students (Table 3). Overall, 20.0% fulfilled the WHO recommendations for PA accumulating ≥60 min of MVPA per day; 16.1% of the girls and 24.5% of the boys (*p* < 0.05) (Fig. 2).

**Table 3:** Physical activity by sex, self-perceived health, SES and study program in 16/17-year-olds. The Tromsø Study: Fit Futures

**Total**		N	MVPA Mean (95% CI)	CPM Mean (95% CI)	Steps Mean (95% CI)	
		611	44.1 (42.4–45.8)	338.2 (328.5–347.8)	7831 (7632–8030)	
**Self-perceived health**	Very bad /bad	34	38.1 (31.2–45.1)	307.4 (267.9–347.0)	7161 (6371–7952)	
	Neither good nor bad	122	42.0 (38.4–45.6)	317.4 (295.9–339.0)	7438 (6994–7882)	
	Good	295	41.7 (39.5–44.0)	326.7 (314.5–339.0)	7741 (7471–8010)	
	Excellent	150	52.0 (47.9–56.0)	385.4 (362.7–408.1)	8469 (8022–8917)	
	**ANOVA statistics**		F 9.4, *p* < 0.01	F 10.7, *p* < 0.01	F 5.3, p < 0.01	
**Parents highest level of education (SES)**	Don’t know	125	42.9 (39.0–46.8)	329.1 (307.8–350.4)	7614 (7187–8041)	
	Primary school 9 years	22	37.7 (29.0–46.3)	309.5 (266.5–352.4)	7546 (6424–8669)	
	Vocational high school	78	39.1 (34.9–43.3)	312.3 (289.4–335.1)	7620 (7101–8138)	
	High school	82	41.9 (37.4–46.5)	330.1 (304.1–356.1)	7791 (7270–8311)	
	College < 4 years	117	45.8 (41.9–49.6)	347.5 (325.0–370.0)	7961 (7480–8443)	
	College ≥4 years	178	47.9 (44.5–51.3)	356.8 (337.1–376.5)	8030 (7638–8423)	
	**ANOVA statistics**		F 2.7, p < 0.05	F 2.1, *p* = 0.06	F 0.6, *p* = 0.67	
**Study program**	Vocational	276	38.5 (36.3–40.7)	309.5 (297.6–321.4)	7359 (7088–7629)	
	General studies	274	44.1 (41.9–46.4)	336.5 (323.2–349.9)	7791 (7506–8076)	
	Sports	61	69.1 (62.3–76.0)	475.1 (435.3–514.8)	10,135 (9441–10,812)	
	**ANOVA statistics**		F 60.4, p < 0.01	F 54.3, p < 0.01	F 34.0, *p* < 0.01	
**BOYS, total**		270	47.6 (44.9–50.4)	353.3 (337.8–368.8)	7853 (7545–8162)	
**Self-perceived health**	Very bad /bad	13	46.4 (32.7–60.1)	334.3 (263.5–405.1)	7866 (6251–9480)	
	Neither good nor bad	60	44.3 (38.3–50.2)	332.3 (295.4–269.3)	7310 (6610–8010)	
	Good	114	45.2 (41.5–49.0)	338.7 (318.4–359.1)	7731 (7265–8196)	
	Excellent	79	53.9 (48.2–59.6)	391.9 (360.2–323.8)	8383 (7819–8946)	
	**ANOVA statistics**		F 2.9, p < 0.05	F 3.5, p < 0.05	F 2.1, *p* = 0.1	
**Parents highest level of education (SES)**	Don’t know	66	48.2 (41.8–54.6)	359.4 (325.9–393.0)	7859 (7179–8538)	
	Primary school 9 years	10	40.5 (26.2–54.7)	296.5 (222.5–370.6)	6990 (4946–9034)	
	Vocational high school	34	39.7 (33.2–46.3)	306.7 (272.7–340.7)	7375 (6544–8205)	
	High school	41	49.2 (42.0–56.5)	370.7 (328.5–412.9)	8380 (7530–9230)	
	College < 4 years	44	51.9 (45.4–58.4)	372.5 (334.0–411.0)	8149 (7445–8852)	
	College ≥4 years	71	48.1 (42.9–53.3)	353.1 (320.5–385.7)	7634 (7031–8237)	
	**ANOVA statistics**		F 1.4, *p* = 0.23	F 1.6, *p* = 0.15	F 1.0, *p* = 0.41	
**Study program**	Vocational	146	44.2 (41.1–47.4)	318.4 (323.3–357.3)	7759 (7363–8155)	
	General studies	90	42.8 (38.9–46.7)	340.3 (295.0–341.7)	7080 (6614–7547)	
	Sports	34	75.2 (65.7–84.7)	501.8 (442.5–561.2)	10,298 (9379–11,217)	
	**ANOVA statistics**		F 36.2, p < 0.01	F 32.5, p < 0.01	F 22.8, p < 0.01	
**GIRLS, total**		341	41.2 (39.1–43.4)	326.2 (314.0–338.3)	7814 (7553–8075)	
**Self-perceived health**	Very bad /bad	21	33.0 (25.5–40.5)	290.8 (240.5–341.1)	6725 (5854–7596)	
	Neither good nor bad	62	39.8 (35.5–44.2)	303.0 (279.6–326.4)	7561 (6988–8134)	
	Good	181	39.6 (36.8–42.3)	319.2 (303.8–334.5)	7747 (7415–8079)	
	Excellent	71	49.8 (43.9–55.7)	378.1 (344.9–411.3)	8568 (7844–9291)	
	**ANOVA statistics**		F 6.2, p < 0.01	F 7.0, p < 0.01	F 3.9, *p* < 0.01	
**Parents highest level of education (SES)**	Don’t know	59	37.0 (33.1–40.8)	295.1 (271.9–318.3)	7340 (6836–7844)	
	Primary school 9 years	12	35.3 (22.9–47.7)	320.3 (260.4–380.1)	8010 (6582–9437)	
	Vocational high school	44	38.6 (32.9–4.2)	316.6 (284.6–348.5)	7809 (7127–8492)	
	High school	41	34.6 (29.8–39.5)	289.5 (262.8–316.2)	7216 (6625–7807)	
	College < 4 years	73	42.0 (37.3–46.8)	332.4 (304.6–360.2)	7850 (7193–8505)	
	College ≥4 years	107	47.8 (43.3–52.2)	359.2 (334.2–384.2)	8293 (7776–8811)	
	**ANOVA statistics**		F 4.2, p < 0.01	F 3.7, p < 0.01	F 1.8, *p* = 0.12	
**Study program**	Vocational	130	32.0 (29.3–34.7)	275.0 (260.3–289.7)	6912 (6558–7265)	
	General studies	184	44.8 (42.0–47.6)	345.4 (329.2–361.6)	8139 (7789–8489)	
	Sports	27	61.5 (51.8–71.1)	441.4 (390.2–492.6)	9910 (8817–11,004)	
	**ANOVA statistics**		F 36.2, p < 0.01	F 35.5, p < 0.01	F 22.9, p < 0.01

Mean total PA (CPM) was 8.3% higher in boys than in girls (p < 0.05). In both boys and girls, PA levels were lower during weekend days compared to weekdays (for girls 12.9% and for boys 15.3% lower on weekends, *p* < 0.001). The only exception to this was boys studying sports, with 13% more CPM during weekend (data not shown). Variations in CPM were greater during weekends (mean CPM 299.4, SD 178.4) than during weekdays (mean CPM 348.3, SD 126.6). Mean CPM increased significantly with better rating of self-perceived health (*p* < 0.05), and with parents’ education for girls (p < 0.05), but not for boys (*p* > 0.05). Participants who attended the sports program had considerably higher means of CPM than the other study programs (p < 0.05, Table 3).

**Fig. 3:**
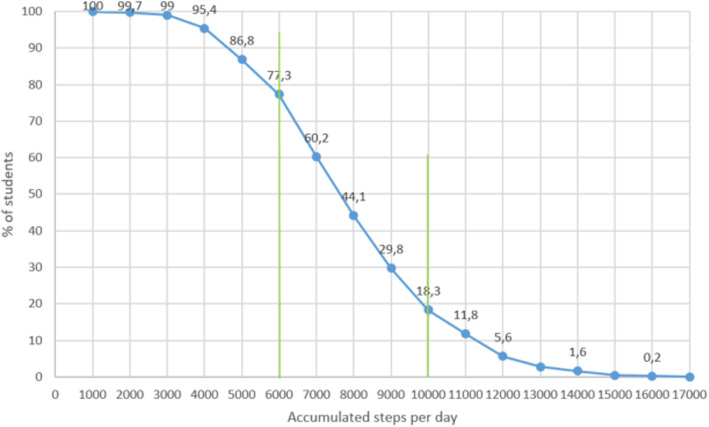
Distribution of mean number of steps per day in 16/17 years old boys and girls, The Tromsø Study -Fit Futures

Mean steps were similar in boys and girls (total steps 7831, 95% CI 7632–8030, Table 3). In total, 18.3% of the participants (15.9% of the girls and 21.3% of the boys) accumulated ≥10,000 steps per day, whereas 76.9% of the participants accumulated ≥6000 steps per day and almost all (99%) accumulated at least 3000 steps per day (Fig. 3).

## Discussion

Our results suggest that approximately 20% of 16–17-year-old boys and girls fulfilled the current WHO recommendations for PA. Boys were more physically active than girls, as they accumulated more minutes in MVPA and higher CPM. However, steps per day were similar between boys and girls. Moreover, both boys and girls had higher mean CPM during weekdays than weekends.

Our results are at large in accordance with other studies assessing PA by accelerometry in adolescents [10, 20–22]. A challenge when comparing different studies of accelerometer measured PA is the lack of standardization of cut-points for intensity categories [20]. For example, the lower cut-point for MVPA ranges from 1000 CPM to 3000 CPM [20], affecting comparison between studies. A cross-sectional study by Ruiz et al. (2011) including nine European countries (the HELENA study) using compatible, although not identical cut-points for MVPA showed that 41% of adolescents (mean age 14.9 years) met the recommended activity levels (27.5% of the girls and 56.8% of the boys) [37]. These proportions are substantially larger than in our study, but the HELENA study included a wider age-span and the sample was somewhat younger than ours. A recent review suggested that the compliance with meeting PA recommendations ranged from 0 to 60%, depending on intensity threshold used [20], emphasizing the need for data harmonization for cross-study comparisons.

The higher activity levels in boys in our study is consistent with previous studies [20, 22, 38]. Even though boys and girls accumulate about the same amount of steps, there is a general agreement that MVPA is essential for health benefits [39], and step counts do not assess the intensity of PA. The difference between girls and boys in this study seems to be more similar to national studies performed on children and adults [9, 10], than to international studies performed on adolescents [20, 37, 40]. Even though there is a statistically significant difference between girls and boys also in the Norwegian studies, the difference is much higher in the international studies. We don’t know why, but perhaps it could be due to a strong gender equality policy in Norway, where parents and school endeavour to give boys and girls an equal upbringing. It is less probable that this is only due to methodological differences, as these are studies done with objective measurements, and accelerometer cut points are similar in the different studies.

We expected the PA levels in our sample of adolescents aged 16–18 years to be lower than in younger children but higher than in adults. However, we found that the mean CPM in our sample was similar as that previously observed in Norwegian adults [9, 41]. Here, a decline in PA of 30% in females and 35% in males between adolescents aged 15 years and adults between 20 and 64 years of age, was found [9]. Although speculative, comparing these results with those from this study, suggests that this decline occurs at the age of 16 to 18 years, when adolescents move from lower secondary school to upper secondary school.

We found lower PA during weekends compared to weekdays, which is in line with previous studies [20, 38]. Also worth mentioning is that the variation is larger during weekends, as some of the adolescents increase their activity.

The positive association between self-perceived health and PA is consistent with the findings in several other studies [42–46]. This is a young and physically healthy population, but despite this we found a significant correlation between the level of PA and self-perceived health status. This study did not investigate causality, and it is therefore not possible to ascertain the direction of this association. Nevertheless, a low level of PA might contribute to a lower health status over time, which again may lead to even less PA.

We have not been able to find other studies comparing levels of PA in different school programs. It might be considered obvious that students in a sports class are more physically active than peers in general studies and vocational studies. This raises the question of whether these students are more active because they are attending a sports study program, or if they attend the sports study program because they lead a more active lifestyle. The two are not mutually exclusive. This study did not differentiate between school time and after school activity. However, several studies imply that increased PA during the school day increases total PA [47–49]. We consider our result to be in accordance with these studies.

### Strengths and weaknesses

We consider the high participation rate and the objective PA measurements as the main strengths of our study. The ActiGraph wGT3X has high validity compared with self-reported PA [50] and compared with other accelerometer devices [51, 52] and is used in several other cohort studies [10, 20, 21, 53]. However, accelerometer measurements have limitations, such as being unable to accurately assess the intensity while graded walking, carrying loads such as groceries or a rucksack, and cycling [54]. Recommendations for PA for both children, adolescents and adults include strength conditioning exercises, and many adolescents tend to shift from team-sports to gym based strength exercising [55], which is not measured accurately by accelerometry [56]. The accelerometer was mounted on the hip with a belt and was removed when sleeping and during water activities. This may increase non-wear time if participants forgot to attach the monitor after these activities. Therefore, continuous 24-h measurements with waterproof equipment are preferable. We chose to use the uniaxial data to be able to compare our results to previous studies. The choice of 60 s epoch will obscure the actual variation in activity, and possibly result in fewer minutes of VPA than if 10 s epochs were used [57].

PA levels tend to fluctuate during the day, week, and between seasons. A limitation of this study is that the measurements were done during one single week, and do not capture seasonal variability. Previous studies have documented lower PA levels during the winter and during periods with poor weather conditions [58–60]. In Norway, and particularly in the northern part with substantial difference in temperature and daylight between winter and summer, it is likely that the seasonal variability affects PA levels. The measurements in our study were conducted between September and May, covering 3 seasons. However, for practical reasons students from the same school and study program were measured during the same period. Although the difference between study programs were as expected, it precludes robust analyses of the influence of season.

## Conclusions

The majority of 16- to 17-year-old adolescents living in Northern Norway do not fulfil the current WHO recommendations for physical activity. Total PA volumes were similar to those reported in Norwegian adults. PA varied with sex, self-perceived health and study program. Inadequate levels of PA is a significant challenge for public health, and efforts should be made to increase PA to recommended levels. Health officials would profit from a future research focus on identifying the least physically active individuals and specifically target these groups for interventions.

## Supplementary information

**Additional file 1.** Overview of questions from questionnaire used in this study. Contains the questions and the response alternatives to each question, translated from Norwegian to English.

## Data Availability

The data that support the findings of this study are available from The Tromsø Study, but restrictions apply to the availability of these data, which were used under license for the current study, and so are not publicly available. Data are available from the The Tromsø Study upon application. To apply for data, please visit the Tromsø Study web page at: https://en.uit.no/forskning/forskningsgrupper/sub?p_document_id=453582&sub_id=71247

## Abbreviations

BMI: Body Mass Index
CPM: Count per minute
MVPA: Moderate to vigorous Activity
PA: Physical activity
SES: Socioeconomic Status
TFF1: Tromsø Study- Fit Futures 1
VM: Vector Magnitude
WHO: World Health Organisation.
