# Supplementary material for: Prevalence of accelerometer-measured physical activity in adolescents in Fit Futures – part of the Tromsø Study
Source: BMC Public Health. 2020 Jul 17;20:1127. doi: 10.1186/s12889-020-09171-w (PMC7368757; doi:10.1186/s12889-020-09171-w)
Supplement: Supplementary file 1 — Additional file 1. Overview of questions from questionnaire used in this study. Contains the questions and the response alternatives to each question, translated from Norwegian to English. [file 12889_2020_9171_MOESM1_ESM.docx]

# Overview of questions used in this study

Do you smoke?

-No, never

-Sometimes

-Daily

Do you have any chronic or persistent disease?

-Yes

-No

If yes, which diagnosis and how old were you when you had this disease the first time?

-Diagnosis 1: Age disease 1:

-Diagnosis 2: Age disease 2:

-Diagnosis 3: Age disease 3:

-

-

How do you in general consider your own health to be?

-Excellent

-Good

-Neither good nor bad

-Bad

-Very bad

What is the highest education completed by your mother?

-Primary school, 9 years

-Occupational high school

-High School

-College less than 4 years

-College 4 years or more

-Don’t know

What is the highest education completed by your father?

-Primary school, 9 years

-Occupational high school

-High School

-College less than 4 years

-College 4 years or more

-Don’t know
